# Mental symptoms and cause-specific mortality among midlife employees

**DOI:** 10.1186/s12889-016-3816-0

**Published:** 2016-11-08

**Authors:** Eero Lahelma, Olli Pietiläinen, Ossi Rahkonen, Jouni Lahti, Tea Lallukka

**Affiliations:** 1 Department of Public Health, University of Helsinki, PO Box 20 , (Tukholmankatu 8 2B), 00014 Helsinki, Finland; 2 Finnish Institute of Occupational Health, and Department of Public Health, University of Helsinki, Helsinki, Finland

**Keywords:** Mental symptoms, GHQ, SF-36, Mortality, Causes of death, Employees

## Abstract

**Background:**

Mental symptoms are prevalent among populations, but their associations with premature mortality are inadequately understood. We examined whether mental symptoms contribute to cause-specific mortality among midlife employees, while considering key covariates.

**Methods:**

Baseline mail survey data from 2000–02 included employees, aged 40–60, of the City of Helsinki, Finland (*n* = 8960, 80 % women, response rate 67 %). Mental symptoms were measured by the General Health Questionnaire 12-item version (GHQ-12) and the Short Form 36 mental component summary (MCS). Covariates included sex, marital status, social support, health behaviours, occupational social class and limiting long-standing illness. Causes of death by the end of 2013 were obtained from Statistics Finland (*n* = 242) and linked individually to survey data pending consent (*n* = 6605). Hazard ratios (HR) and 95 % confidence intervals (95 % CI) were calculated using Cox regression analysis.

**Results:**

For all-cause mortality, only MCS showed a weak association before adjustments. For natural mortality, no associations were found. For unnatural mortality (*n* = 21), there was a sex adjusted association with GHQ (HR = 1.96, 95 % CI = 1.45–2.64) and MCS (2.30, 95 % CI = 1.72–3.08). Among unnatural causes of death suicidal mortality (*n* = 11) was associated with both GHQ (2.20, 95 % CI = 1.47–3.29) and MCS (2.68, 95 % CI = 1.80–3.99). Of the covariates limiting long-standing illness modestly attenuated the associations.

**Conclusions:**

Two established measures of mental symptoms, i.e. GHQ-12 and SF-36 MCS, were both associated with subsequent unnatural, i.e. accidental and violent, as well as suicidal mortality. No associations were found for natural mortality due to diseases. These findings need to be corroborated in further populations. Supporting mental health through workplace measures may help counteract subsequent suicidal and other unnatural mortality among midlife employees.

## Background

Mental disorders have serious consequences for the sufferers and major contributions to the burden of disease and mortality [1, 2]. It is estimated that people with mental disorders may lose 10 years of potential life, and that up to 14 % of deaths worldwide, i.e. each year eight million deaths, involve people with mental disorders [3, 4]. Such excess mortality risk concerns those with severe and diagnosed mental disorders, including schizophrenia, bipolar disorder, major depression and psychoses [3]. However, people do not die from these disorders. Most deaths are due to natural mortality from chronic diseases, such as cardiovascular diseases, cancers and infections and a smaller part is due to unnatural non-disease causes, including accidents and violence. Among people with mental disorders, suicide is an important unnatural cause of death [3–5].

Evidence on the associations between mental health and mortality comes primarily from studies examining diagnosed mental disorders among patients. Much less is known about non-diagnosed mental problems, which include milder and self-reported symptoms of e.g. depression, anxiety and worthlessness [6–8]. Quantitative estimates for mental symptoms are provided by established measures like the General Health Questionnaire (GHQ) [9] and the Short Form 36 (SF-36) mental component summary (MCS) [10].

Mental symptoms are prevalent among general and employed populations, and depending on the measure and cut-off point, up to 15–30 % suffer from such disorders across various national contexts [11–14]. Mental symptoms impair people’s well-being, social functioning and work ability, signal the need for treatment and may eventually lead to early exit from work [15–17]. As severe and diagnosed mental disorders are known to convey a risk of premature death [3], it is justified to ask whether the same concerns also milder and self-reported mental symptomatology, which is prevalent in unselected populations.

A number of studies on the associations between mental symptoms and mortality have used clinical samples, specific disease groups or elderly people. Some studies have also used unselected general or employed populations. In a representative sample of British adults, mental symptoms were associated with all-cause, cardiovascular and cancer mortality, with the strongest association for unnatural mortality [18]. Higher GHQ scores implied greater mortality risks in a dose–response manner, and the risks remained after adjusting for sociodemographic and health-related factors. Other studies on British general populations have equally reported an association between mental symptoms and all-cause [19–22], cardiovascular and respiratory mortality [23, 24]. Some studies have observed sex differences, with stronger associations for men than women [19–22]. A further study found that cardiovascular mortality risk was particularly high for those with highest GHQ scores, and low social class strengthened the risk [25]. After considering sociodemographic and health-related factors, the associations between mental symptoms and mortality typically attenuate [18, 20, 24] and may even become non-existent [19, 21].

While GHQ has been the dominant measure, SF-36 MCS has also been used. In a Scottish general population, MCS was associated with all-cause mortality but no longer after considering health-related covariates [26]. Among elderly people from Taiwan, MCS remained weakly associated with all-cause mortality even after adjustments [27]. In a US study on middle aged and elderly women [28], and in a Spanish study on elderly people [29], both stability and major increase in mental symptoms measured by MCS conveyed a mortality risk before and after adjustments.

The prior research suggests that also milder and self-reported mental symptoms are associated with subsequent all-cause and natural mortality. However, the associations tend to be relatively weak, dependent on covariates or even non-existent [16–26, 30]. To be able to unravel possible underlying associations between mental symptoms and mortality, further cause-specific analyses are needed. Limited attention has been devoted to both natural and unnatural mortality, and the importance of accidental and violent causes has remained undiscovered. Only rarely has more than one single measure of mental symptoms been included to strengthen the reliability and validity of the findings. So far, the associations between mental symptoms and subsequent mortality, in particular from unnatural causes, are inadequately understood.

Our aim was to examine the associations between mental symptoms, as indicated by both GHQ and SF-36 MCS, and subsequent mortality due to any cause, natural causes and unnatural causes. This is done among Finnish midlife employees, while considering sociodemographic and health-related covariates.

## Methods

### Baseline data

The data were derived from the Helsinki Health Study cohort on the staff of the City of Helsinki. The municipality is the largest employer in Finland, with almost 40 000 white-collar and blue-collar employees in hundreds of different occupations. The municipality provides basic services, including health and social welfare, education and culture, technical maintenance, public transportation and general administration [31].

The baseline mail surveys were collected in 2000, 2001 and 2002 among employees who reached the ages of 40, 45, 50, 55 and 60 in each year. The original sample included 13 344 employees who received questionnaires. At baseline 8 960 employees participated (response rate 67 %, 80 % women). The sex distribution corresponds to the staff of the City of Helsinki and the Finnish municipal sector in general. Non-response analyses showed that the baseline respondents broadly represent the target population, with men, younger employees, manual workers and those with prolonged sickness absence being slightly underrepresented [31].

### Mortality

Causes of death (ICD-10) were obtained from Statistics Finland registers. Mortality was followed up from responding to the baseline survey in 2000–2002 until the end of 2013. The mean follow up time was 12.5 years. Deaths were individually linked to the survey data for those who consented to the linkage (74 % of the respondents). The number of participants analysed in this study was 6605 (78 % women). Altogether 242 (159 women and 83 men) participants died over the follow up (Table 1). Non-response analyses also examined the effect of consenting to data linkage and suggested that bias by sociodemographics and sickness absence was minor [31].

**Table 1:** Participants and mortality by cause of death

	Participants		All-cause mortality		Natural mortality		Unnatural mortality			
							All		Suicides	
	*n*	%	*n*	%	*n*	%	*n*	%	*n*	%
Women	5185	79	159	66	149	67	10	(49)	6	(52)
Men	1420	21	83	34	72	33	11	(51)	5	(48)
All	6605	100	242	100	221	100	21	(100)	11	(100)

### Mental symptoms

Mental symptoms were derived from the baseline surveys and measured by the General Health Questionnaire 12-item version (GHQ-12) [9] as well as the Short Form 36 (SF-36) mental component summary (MCS) [10].

GHQ is a general inventory of mental symptoms with reference to the recent past. It includes symptoms of depression, anxiety, sleep, worthlessness and happiness, with scores ranging from 0 to 12, but does not indicate particular diagnoses. Higher scores signal the presence of mental symptoms. The proportion of those with GHQ score 3–12 was 24 %. According to validation studies, GHQ is predictive of the need for treatment and onset of more severe mental disorders [7, 32]. The Cronbach alpha for internal consistency in these data was 0.91.

MCS was derived from 36 questions of the SF-36 inventory, which depicts eight subdomains of health. MCS includes symptoms from the subdomains of vitality, social functioning, emotional roles and mental health. MCS and GHQ share similar mental symptoms, with MCS having additional emphasis on functioning and quality of life. The MCS scores range from 0 to 100, and neither indicate particular diagnoses. Lower scores signal the presence of mental symptoms. MCS is scaled to have a mean of 50 and standard deviation of 10 in the general US population. SF-36 is a reliable and well-validated measure of health [10, 33].

### Covariates

There were no sex interactions in the studied associations and we pooled men and women in the analyses. In previous studies occupational social class [21, 34], social support and relations [34, 35], health behaviours [21, 34] and physical and general ill health [19, 21] have had an impact on the association between mental health and mortality. We included similar variables as covariates. Marital status was categorised into married/cohabiting and unmarried including divorced and widowed. Social support [36] was categorised into low, intermediate and high support. Occupational social classes were managers and professionals, semi-professionals, clerical employees and manual workers. Smoking was categorised into never smokers, ex-smokers and current daily smokers. Drinking problems were measured by CAGE (Cut-down, Annoyed, Guilt, Eye-opener) inventory [37], with scores 3–4 indicating drinking problems and scores 0–2 no problems. Body mass index (BMI) was calculated from self-reported weight and height and used as a continuous variable. Physical activity was measured by metabolic equivalent values (MET) and used as a continuous variable [38]. Limiting long-standing illness was categorised into those with and those without such illness.

### Statistical methods

Multiple imputation on the studied variables was used to create ten datasets with missing values replaced by the imputed ones [39]. The analyses were then run on each imputed dataset and the results combined by taking averages of the coefficients and adjusting standard errors for the imputation. The data were assumed missing at random.

Both GHQ and MCS were used as continuous scores. The scores were standardised by subtracting the sample mean score from the individual score and dividing by the sample standard deviation. For GHQ-12, the mean score was 1.92 and the standard deviation 3.12. For SF-36 MCS the mean score was 51.57 and the standard deviation 9.83.

Cox proportional hazards models were fitted to yield hazard ratios (HR) and their 95 % confidence intervals (95 % CI). Reference is made to the mean score of GHQ and MCS, and the HRs indicate the risk of mortality at the distance of one standard deviation from the mean. Age was used as time axis rendering age adjustment redundant. Model 1 adjusted for sex; Model 2 sex, marital status and social support; Model 3 sex and occupational social class; Model 4 sex and health behaviours (smoking, CAGE, MET, BMI); and Model 5 sex and limiting long-standing illness. Model 6 was a full model adjusting for all covariates simultaneously. R statistical software version 2.13.0 was used.

## Results

Of the 242 deaths 221 (91 %) were from diseases, i.e. natural mortality (Table 2). The major causes of death were cancer (*n* = 132), cardiovascular (*n* = 48) and gastrointestinal diseases (*n* = 17). Unnatural mortality included suicides (*n* = 11) and accidents (*n* = 10).

**Table 2:** Associations^a^ between mental symptoms (GHQ-12) and mortality. Men and women pooled (*n* = 6605)

	Model 1^b^ HR 95 % CI	Model 2 HR 95 % CI	Model 3 HR 95 % CI	Model 4 HR 95 % CI	Model 5 HR 95 % CI	Model 6 HR 95 % CI
All-cause mortality	1.06 (0.94, 1.20)	1.06 (0.94, 1.19)	1.07 (0.95, 1.21)	1.00 (0.89, 1.13)	1.02 (0.90, 1.15)	0.98 (0.86, 1.11)
Natural mortality	0.97 (0.84, 1.11)	0.96 (0.84, 1.10)	0.97 (0.85, 1.12)	0.91 (0.79, 1.05)	0.93 (0.81, 1.07)	0.90 (0.78, 1.04)
Unnatural mortality	1.96 (1.45, 2.64)	1.88 (1.39, 2.54)	1.96 (1.45, 2.64)	1.82 (1.33, 2.48)	1.75 (1.28, 2.40)	1.61 (1.17, 2.22)
Suicidal mortality	2.20 (1.47, 3.29)	2.09 (1.39, 3.15)	2.17 (1.46, 3.23)	2.13 (1.41, 3.21)	1.88 (1.24, 2.87)	1.71 (1.11, 2.63)

^a^Hazard ratios (HR) and their 95 % confidence intervals (95 % CI) from Cox regression analysis with age as time axis. Models

^b^Models: Model 1 (M1) = + sex; Model 2 = M1 + marital status + social support; Model 3 = M1 + social class; Model 4 = M1 + drinking problems, smoking, physical exercise, BMI; Model 5 = M1 + limiting long-standing illness; Model 6 = + all covariates

### GHQ and mortality

GHQ-12 was unassociated with all-cause mortality as well as natural mortality in any model. However, GHQ was associated with unnatural mortality in the sex-adjusted model 1 (HR 1.96, 95 % CI 1.45–2.64) (Table 2). Of the covariates, limiting long-standing illness in model 5 attenuated the association by 11 % compared to model 1. The effects of the other covariates were weaker and the association between GHQ and unnatural mortality remained even after full adjustment in model 6.

Within unnatural causes, GHQ was associated with suicidal mortality in the sex-adjusted model 1 (HR 2.20, 95 % CI 1.47–3.29). Limiting long-standing illness in model 5 attenuated the association by 15 % and the association remained even after full adjustment in model 6.

### MCS and mortality

SF-36 MCS was weakly associated with all-cause mortality in the sex-adjusted model 1 (HR 1.18, 95 % CI 1.06–1.32) as well as in models 2, 3 and 5, but unassociated after full adjustment in model 6 (Table 3). MCS was unassociated with natural mortality in any model. In accordance with GHQ, also MCS was associated with unnatural mortality in the sex-adjusted model 1 (HR 2.30, 95 % CI 1.72–3.08) and the association remained even after full adjustment in model 6.

**Table 3:** Associations^a^ between mental symptoms (SF-36 MCS) and mortality. Men and women pooled (*n* = 6605)

	Model 1^b^ HR 95 % CI	Model 2 HR 95 % CI	Model 3 HR 95 % CI	Model 4 HR 95 % CI	Model 5 HR 95 % CI	Model 6 HR 95 % CI
All-cause mortality	1.18 (1.06, 1.32)	1.17 (1.05, 1.31)	1.20 (1.07, 1.34)	1.11 (0.98, 1.24)	1.17 (1.04, 1.31)	1.09 (0.97, 1.23)
Natural mortality	1.07 (0.95, 1.22)	1.07 (0.94, 1.21)	1.09 (0.96, 1.23)	1.00 (0.88, 1.14)	1.07 (0.94, 1.21)	1.00 (0.88, 1.14)
Unnatural mortality	2.30 (1.72, 3.08)	2.23 (1.66, 3.01)	2.31 (1.73, 3.09)	2.17 (1.60, 2.95)	2.12 (1.56, 2.87)	1.95 (1.41, 2.69)
Suicidal mortality	2.68 (1.80, 3.99)	2.63 (1.72, 4.04)	2.72 (1.82, 4.07)	2.66 (1.74, 4.08)	2.45 (1.60, 3.75)	2.28 (1.43, 3.64)

^a^Hazard ratios (HR) and their 95 % confidence intervals (95 % CI) from Cox regression analysis with age as time axis

^b^Models: Model 1 (M1) = + sex; Model 2 = M1 + marital status + social support; Model 3 = M1 + social class; Model 4 = M1 + drinking problems, smoking, physical exercise, BMI; Model 5 = M1 + limiting long-standing illness; Model 6 = + all covariates

Similarly in accordance with GHQ, MCS was even more strongly associated with suicidal mortality in the sex-adjusted model 1 (HR 2.68, 95 % CI 1.80–3.99) and the association remained even after full adjustment in model 6.

## Discussion

We sought to find out whether mental symptoms, as indicated by the General Health Questionnaire 12-item version and the Short Form 36 mental component summary, are associated with subsequent all-cause and cause-specific mortality in a cohort of midlife employees from Finland. Our main findings were:

1. Neither measure of mental symptoms was associated with all-cause or natural mortality after adjustments.
2. Both measures of mental symptoms were associated with unnatural mortality.
3. The association between mental symptoms and unnatural mortality was strongest for deaths by suicide.
4. The associations for suicidal and other unnatural mortality remained even after full adjustment for sociodemographic and health-related covariates.

### Comparison and interpretation

Those with severe and diagnosed mental disorders are less likely to be employed [40–42]. However, milder and self-reported mental symptoms are prevalent also among employed people, with repercussions on their functioning, work ability and even mortality [4]. In our study, all-cause mortality, after considering covariates, showed no associations with either prior GHQ or MCS. Disease-based natural mortality neither showed any associations with GHQ or MCS.

In some previous studies, associations between similar mental symptoms and all-cause mortality have been found before and after considering sociodemographic and health-related covariates [18, 20, 23, 25], whereas in some others the associations have not survived adjustments for prior ill health [19, 21]. The associations for all-cause mortality have been relatively weak and observed, in particular, for the highest scores of mental symptom scales [18, 23]. Examining mental symptoms longitudinally, their stability and adverse changes have shown associations with all-cause mortality [28, 29]. We measured mental symptoms at baseline and the stability and remission over the follow up remains an open question. In a British study, social class was a modifier and the lowest class had the greatest mortality risk due to mental symptoms [25], whereas in our study social class had no effects on the studied associations. Future studies would benefit from measuring symptomatology longitudinally as well as considering a broad range of sociodemographic and health related covariates.

Mental symptoms have further shown associations with cardiovascular, respiratory, liver disease and cancer mortality [18–21, 23, 43 ]. These associations have been confirmed primarily for the highest scores of mental symptoms. The associations have been relatively weak and they have attenuated or even rendered non-existent after considering sociodemographics and prior ill health.

In our study, the association of mental symptoms with unnatural mortality was strong and consistent before and after adjustments. Half of the unnatural deaths were accidental, such as traffic accident, drowning or poisoning. However, the evidence of an association between mental symptoms and mortality from unnatural causes is limited [18, 23, 44], with one study finding no association for mortality from injuries [20]. Substance abuse, in particular alcohol abuse, is related to accidental deaths, and to mental symptomatology [45].

The other half of unnatural deaths was due to suicides that we found to be particularly strongly associated with mental symptoms. Overlap between accidents and suicide is possible, but unknown to us in these data. We acknowledge that the number of unnatural deaths in our study was limited. Nevertheless, the models converged and the associations remained stable and statistically significant throughout the analysis for unnatural mortality in general and suicidal mortality in particular. Similar associations were found for GHQ-12 as well as SF-36 MCS before and after adjustments. While suicide has been previously studied as a cause of death among those with severe and diagnosed mental disorders [4, 23, 44], our understanding of the links between mental symptomatology and subsequent suicide has been much poorer.

That the two instruments of mental symptoms were in a similar way associated with accidental and suicidal mortality provided stronger evidence than using one instrument only. Previous studies have not included simultaneously multiple measures of mental symptoms, and thus comparing the mutual consequences for natural, accidental and suicidal mortality of symptomatology measured in two or more ways has not been possible. Further studies with multiple measures of mental symptoms and mortality are warranted.

Among the risk factors for suicide, prior severe mental disorders play an important role [35, 46, 47]. Of such disorders, depression is the most prevalent among people who die by suicide [5]. The evidence comes largely from clinical samples and uses diagnostic information on mental disorders. Major depression and other diagnosed mental disorders contributing to suicide mortality suggest the importance of mental pathways to suicide. The strong associations between the two instruments of mental symptoms and subsequent suicide mortality suggest that the instruments are markers of such pathways. Thus, mental symptoms are likely related to various comorbid mental disorders, such as depression, anxiety, psychotic and personality problems as well as substance abuse. It has been shown that mental symptoms, such as those indicated by GHQ, are comorbid, in particular, with depressive and anxiety disorders [48]. In order to provide a fuller picture of the contribution of mental symptomatology to suicide and other unnatural causes of death, further studies need to consider multiple comorbid disorders in conjunction with mental symptoms.

The current evidence on the associations between mental symptoms and mortality is based on a limited number of studies using divergent study populations, sample sizes, study designs, measures of mental symptoms, causes of death and sets of covariates. This contributes to the heterogeneity emerging from the existing research base, showing both associations and no associations. Our study, suggesting associations between mental symptoms and unnatural mortality, in particular suicidal mortality, highlights the need for valid measures and strong study designs.

### Methodological considerations

Participation to our baseline survey was acceptable. Consenting to data linkage was high but contributed further to the non-participation. We were able to conduct extensive non-response analyses for both participation and consenting to data linkage [31]. These analyses suggest that major bias in the data is unlikely. However, we acknowledge that the non-participation remains a potential source of bias.

An advantage was that reliable and complete register based data on causes of death could be individually linked to our baseline survey data, containing a range of sociodemographic and health-related covariates. Nevertheless, residual confounding cannot be ruled out.

We were able to employ two often-used instruments of mental symptoms, i.e. the General Health Questionnaire [9] and the Short Form 36 mental component summary [10]. Both are reliable and well-validated inventories, but we acknowledge that self-reports are subject to potential reporting bias, and clinical and diagnostic tools are equally needed. Our instruments reflect generic mental symptomatology and we were unable distinguish between key types of mental symptoms. We were also unable to consider the comorbidity of mental symptoms with more severe mental disorders.

The 12.5-year follow up among employees included relatively few deaths, and the number of unnatural deaths and suicides was limited. Irrespective of this, our analysis was able to produce meaningful and stable estimates, but analyses that are more detailed could not be done.

Our data were derived from a cohort of Finnish midlife employees. The data were thus non-clinical and not based on self-selection. Nevertheless, occupational cohorts tend to be healthier than general populations due to the healthy worker effect. Such selection may well concern our cohort, although milder mental symptoms are less likely to cause exit from work than more severe clinically significant mental disorders.

The female majority in our cohort is a potential limitation as there are sex differences in the causes of death among the working age population, with women dying more often from cancers and men more often from cardiovascular and accidental causes. However, in this midlife employee cohort, male and female mortality was relatively similar and we found no sex interactions in the studied associations between mental symptoms and mortality. Nevertheless, direct generalisations of our findings to broader age groups, general populations or further national contexts are not warranted.

## Conclusions

Our study examined mental symptoms and subsequent all-cause and cause-specific mortality. We found mental symptoms, as indicated by the General Health Questionnaire and the Short Form 36 mental component summary, to be antecedents of unnatural, i.e. accidental and, even more so, suicidal mortality, but not natural mortality. Our findings provided novel evidence and raised novel questions aimed at sharpening the picture on the contribution of mental symptomatology to cause-specific mortality. The findings need to be corroborated in further cohorts including comorbid and diagnostic data, and larger numbers of deaths.

Although life expectancy within the general population is increasing, there is a disparity for people with mental disorders [44]. Supporting employee mental health through workplace measures may help counteract suicidal and other unnatural mortality. Employers, personnel management and occupational health care are in a key position to prevent ill health and work disability as well as providing support and assistance for midlife employees suffering from mental symptoms as well as more severe mental disorders.

## Abbreviations

BMI: Body mass index
CAGE: Cut-down, annoyed, guilt, eye-opener
CI: Confidence interval
GHQ: General Health Questionnaire
HR: Hazard ratio
ICD-10: International Statistical Classification of Diseases and Related Health Problems 10th Revision ICD
MCS: Mental component summary
MET: Metabolic equivalent
PCS: Physical component summary
SF: Short Form
US: United States
